# Laryngeal Chondroma: A Rare Diagnosis in This Localization

**DOI:** 10.1155/2011/852396

**Published:** 2011-11-30

**Authors:** Ebru Tastekin, Semsi Altaner, Cem Uzun, Ahmet R. Karasalihoglu, Cigdem Ozdemir, Ali Kemal Kutlu

**Affiliations:** ^1^Department of Pathology, Trakya University Medical Faculty, 22030 Edirne, Turkey; ^2^Department of Otolaryngology, Trakya University Medical Faculty, 22030 Edirne, Turkey; ^3^Department of Pathology, Tekirdag State Hospital, 22030 Tekirdag, Turkey

## Abstract

Primary chondroid tumors of the larynx represent less than 1% of all laryngeal tumors. Most of them are chondromas and they often involve to the cricoid cartilage. A 31-year-old male patient applied to the oto-laryngology service with a history of dysphonia and dyspnea. Microlaryngoscopy revealed 2 cm sized, ill-defined, covered with regular mucosa, porous, and hard mass on posterior surface of crycoid cartilage in subglottic area. Following the excision of the lesion, histopathologic examination revealed as chondroma. Two years later, local recurrence was detected and the diagnosis was again chondroma. There was no complaint of the patient in last 3 and half years of follow-up. Chondroma should carefully be differentiated from chondrosarcoma and the patients should be followed for possible recurrences.

## 1. Introduction

Cartilaginous tumors of larynx are very rare. Primary chondroid tumors of larynx are less than 1% of all laryngeal tumors [1–3]. But they are most common mesenchymal tumors of larynx [4, 5]. 70–75% of these tumors detect on endolaryngeal part of cricoid cartilage [1–3, 6]. Clinical symptoms are variable and associated with tumor size and localization. Usually stridor, snoring, dyspnea, and neck mass are seen [6].

The incidence of malignancy for chondroid tumors is higher in old patients. However chondromas were seen in 10-year younger patients than chondrosarcomas. Male : female ratio is 1 : 4 [7–11]. Although there is no definitive etiologic factor for these tumors, most commonly known is irregular ossification of the laryngeal cartilage [9–12]. Other etiologic factors are radiotherapy and teflon injection [10]. Macroscopic and microscopic appearances of laryngeal chondromas are similar to chondromas of other sites. 

Cartilaginous tumors of larynx are classified in 4 groups [13]: (1) metaplastic cartilaginous nodules (chondrometaplasia), (2) chondromas, (3) chondrosarcomas, and (4) cartilaginous material is in other neoplasias (pleomorphic adenoma, chondroblastic osteosarcoma etc.). The differential diagnosis of these 4 types is very important.

## 2. Case Report

### 2.1. Clinical Features and First Operation

A 31-years-old male patient was referred to the Chest Medicine Department with a history of dysphonia and dyspnea. Laryngeal subglottic mass was detected with bronchoscopic evaluation. The patient was consulted to the Department of Otolaryngology. 2 cm sized, ill-defined, porous, and hard mass on posterior surface of cricoid cartilage in subglottic area was detected in microlaryngoscopic evaluation. This mass was covered by regular mucosa. This lesion was scraped from posterior wall of cricoid cartilage with sickle scalpel. Mitomycine was applied to the lesion locally to prevent the development of postoperative scar. Clinically tracheostomy was not required and no complication was seen. He was discharged next day.

### 2.2. Pathologic Findings

**Figure 1:**
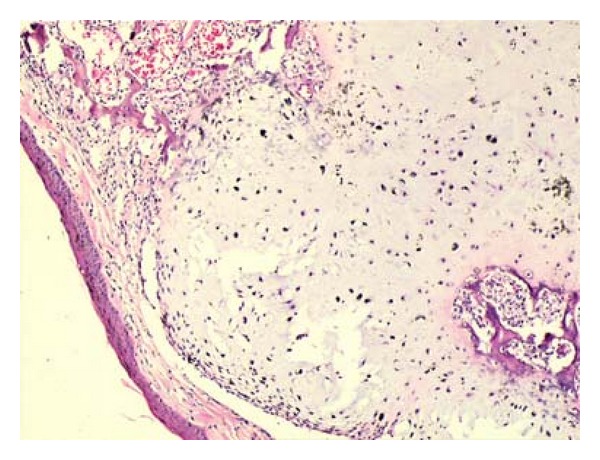
Lobulated tumor near the calcified normal chondroid tissue. Hematoxylen Eosin stain ×50.

**Figure 2:**
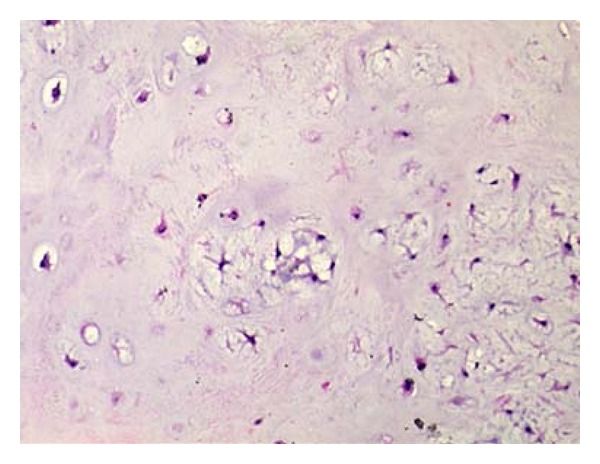
Hypocellular and homogenous appearance of tumor. Tumor cells are small, oval-round shaped (no mitosis and atypia). Hematoxylen Eosin stain ×100.

Macroscopically total volume of 2 cc, 0.2–0.5 cm sized, fragmented, gray-pink colored, medium-hardness, homogenous materials were seen. Microscopically well-defined, lobulated-contoured mass was seen near of the focally calcified cartilaginous tissue (Figure 1). The tumor was very hypocellular and homogenous. Tumor cells were monotone and had small oval-round nuclei. Mostly 20 nuclei were counted in one HPF. Mitosis, necrosis, hemorrhage, and binucleation were not detected (Figure 2). The patient was diagnosed as “Laryngeal chondroma” with these morphologic findings.

### 2.3. Second Operation and Clinical Follow-Up

This patient was referred to the Department of Otolaryngology with stuck feeling in the throat after 2 years from first operation. 1 × 0.8 cm sized, hard, gray-coloured mass, which is covered by regular mucosa, was detected in same localization (right subglottic area). Second microlaryngoscopy was applied and the lesion was excised in november 2007. This lesion was diagnosed as “laryngeal chondroma” too. In routine follows-up no complaints and lesion were detected (42 months).

## 3. Discussion

Chondroid tumors of the larynx are uncommon neoplasms and they are comprised up to 1% of all laryngeal neoplasms. However they are most common mesenchymal tumors of larynx [14]. Some studies supported that the incidence of malign chondroid tumors is higher than benign types [7–12, 15]. Chondroma and low-grade chondrosarcoma are most common types [14]. These tumors are localized in posterior lamina of cricoids cartilage [1–6, 14]. Malign forms generally are seen in old and male patients [7–10]. The most common symptoms are dyspnea, hoarseness, and compression to the neighbor tissues.

Chondroid tumors of larynx can be easily recognized histologically, because of their characteristic features. But morphologic features must be well known for differential diagnosis between subtypes.

Chondrometaplastic nodules are smaller than 1 cm sized and multiple. This lesion is characterized by loose of lobular pattern, mucochondroid changes in soft tissue matriks, and well-defined nodules [8–12, 15]. Our patient had a single and 2 cm sized nodule.

Differential diagnosis between chondromas and chondrosarcomas can be difficult. Macroscopically chondromas are smaller than 2 cm (average 1.5 cm) but chondrosarcomas are generally larger than 3 cm sized (average 4.3 cm) [14]. Microscopically chondromas are hypocellular (30–40 nuclei/HPF) lesions with homogenous lobular growing pattern. They have no nuclear atypia and mitosis.

Increased cellularity (more than 40 nuclei/HPF), nuclear atypia, binucleation, mitosis, and pleomorphism are variable in low- and high-grade chondrosarcomas. Our lesion was characterized by hypocellularity, no nuclear atypia, mitosis, binucleation, and pleomorphism. We diagnosed this lesion as “laryngeal chondroma” with these morphologic findings. Our patient referred to the Department of Otolaryngology with recurrent mass in same localization after 2 years. Microlaryngoscopic resection of lesion was diagnosed as “chondroma” too. Routine follows-up (42 months) of patient have been normal.

Chondromas are rare mesenchymal tumors of larynx. They are benign lesions but incidence of locally recurrence is very high. Histopathologicly, differential diagnosis of laryngeal chondromas should be planned very carefully. Especially differential diagnosis between chondromas and low-grade chondrosarcomas is important for planning of treatment.

Laryngeal chondroma was described in a few numbers of reports.

We suggested that the differential diagnosis of chondromas must be done carefully and the follows-up of patients must be planned more frequently. Our case had typical clinical, histopathological, and prognostic features of laryngeal chondromas.
